# Análisis del absentismo en consultas externas de otorrinolaringología desde la perspectiva del paciente

**DOI:** 10.23938/ASSN.1166

**Published:** 2026-08-14

**Authors:** Aurora Lopez-Llames, Paula Hernández-Ruiz, Vicente García-Román, Elena Ronda-Perez

**Affiliations:** 1 Hospital Universitario de Torrevieja Servicio de Otorrinolaringología Torrevieja Alicante España; 2 Hospital Universitario de Torrevieja Servicio de Medicina Preventiva Torrevieja Alicante España; 3 Universidad de Alicante Grupo de investigación en Salud Pública Alicante España

**Keywords:** Pacientes no Presentados, Otorrinolaringología, Servicio Ambulatorio en Hospital, Accesibilidad a los Servicios de Salud, Entrevistas como asunto, No-Show Patients, Otolaryngology, Outpatient Clinics, Hospital, Health Services Accessibility, Interviews as Topic

## Abstract

**Fundamento:**

El absentismo en consultas externas hospitalarias compromete la continuidad asistencial y la eficiencia organizativa, especialmente en especialidades de alta demanda. Este estudio analiza los motivos de inasistencia autodeclarados por pacientes de consultas externas de Otorrinolaringología (ORL), los clasifica según su potencial evitabilidad y evalúa la frecuencia de reprogramación posterior.

**Metodología:**

Estudio transversal realizado en consultas externas de ORL de un hospital público de segundo nivel. De las 1.117 consultas no atendidas registradas durante el año 2024, se seleccionaron aleatoriamente 299. Entre febrero y junio de 2025 se realizaron entrevistas telefónicas estructuradas. Los motivos se clasificaron según tipología predefinida y se agruparon en evitables y no evitables. Se aplicó análisis descriptivo y pruebas χ² y U de Mann-Whitney. Las respuestas abiertas se examinaron mediante análisis temático descriptivo y se vincularon con las dimensiones del modelo de Levesque.

**Resultados:**

La muestra final incluyó 232 consultas (77,5%). El fallo en la comunicación fue el motivo de inasistencia más frecuente (32,3%) seguido de conflictos laborales o personales (19,4%). El 42,2% de las inasistencias se consideraron potencialmente evitables y solo el 25,0% fueron reprogramadas; ninguna de las dos características se relacionó con edad, sexo o nacionalidad. La dimensión *approachability* concentró el 44,6 % de las menciones registradas.

**Conclusiones:**

El absentismo en consultas externas de ORL obedece a causas modificables, vinculadas principalmente a fallos en la comunicación durante la fase inicial del proceso de citación. A pesar de las citas perdidas, los pacientes solo solicitaron la reprogramación posterior de una cuarta parte de las mismas.

## Abstract (en)

**Background:**

Non-attendance at hospital outpatient clinics disrupts continuity of care and reduces healthcare efficiency, particularly in high-demand specialties. This study analyzes patient-reported reasons for non-attendance at otola-ryngology (ENT) outpatient clinics, classifies them according to their potential avoidability, and assesses the frequency of subsequent appointment rescheduling.

**Methods:**

A retrospective observational study was conducted at a secondary-level public hospital. Among 1,117 missed outpatient appointments recorded in 2024, a random sample of 299 was selected. Structured telephone interviews were carried out between February and June 2025. Reasons for non-attendance were categorized using a predefined classification and grouped as avoidable or unavoidable. Descriptive analyses were performed, together with χ² and Mann-Whitney U tests. Open-ended responses underwent descriptive thematic analysis and were mapped to the dimensions of Levesque’s access-to-care framework.

**Results:**

The final analyses included 232 appointments (77.5%). Failure to receive appointment notification was the most frequently reported reason for non-attendance (32.3%), followed by work-related or personal scheduling conflicts (19.4%). Overall, 42.2% of missed appointments were considered potentially avoidable, whereas only 25.0% of them were subsequently rescheduled. Neither avoidability nor rescheduling was associated with age, sex, or nationality. The approachability dimension accounted for 44.6% of all reported reasons.

**Conclusions:**

Non-attendance at ENT outpatient clinics is largely attributable to potentially modifiable factors, particularly failures in the appointment notification process. Despite missed appointments, only one quarter of patients subsequently rescheduled their appointment.

## INTRODUCCIÓN

El absentismo del paciente en las consultas médicas constituye un problema relevante para los sistemas de salud, ya que compromete la eficiencia de los servicios, la continuidad asistencial y el acceso oportuno a la atención^1^^,^^2^. La no asistencia a la cita programada implica una infrautilización de recursos y contribuye al incremento de las listas de espera, dado que ocasiona la recitación posterior de un alto porcentaje de esos pacientes^3^.

El absentismo se asocia a un aumento de la morbilidad, menor adherencia al tratamiento y retrasos diagnósticos^4^^,^^5^. Algunos estudios sobre absentismo han intentado clasificar los motivos de inasistencia en evitables o no evitables, según la capacidad de intervención que tendría el sistema sanitario^3^^,^^6^. Sin embargo, el absentismo también puede interpretarse como el resultado de una inadaptación del sistema de salud a las necesidades del paciente, más que como un simple acto de acudir o no acudir a una cita^7^.

Los modelos teóricos contemporáneos de acceso a la salud, como el propuesto por Levesque y col^8^, describen el acceso a la atención sanitaria como una secuencia dinámica que integra las dimensiones de la accesibilidad con las capacidades del propio paciente. Estas incluyen la capacidad de percibir la necesidad de atención (*approachability*), buscar servicios aceptables (*acceptability*), alcanzar el punto de atención (*availability and accommodation*), asumir los costes asociados (*affordability*) y participar en una atención clínicamente adecuada (*appropriateness*)^8^. Desde esta perspectiva conceptual, la inasistencia puede interpretarse como la interrupción de alguna de estas fases^9^.

El absentismo adquiere particular relevancia en las consultas externas de Otorrinolaringología (ORL), al tratarse de una especialidad con alta demanda y actividad ambulatoria. Las tasas de absentismo informadas muestran una considerable heterogeneidad (entre el 8% y el 40%)^10^^,^^11^, que refleja indirectamente su carácter multifactorial y la influencia de factores sociodemográficos, organizativos y clínicos.

Con el objetivo de identificar esos factores determinantes, los estudios realizados sobre el absentismo emplean bases de datos asistenciales administrativas (registros de citación, agendas electrónicas o sistemas de información hospitalarios) y, en menor medida, historias clínicas electrónicas. Entre los factores más frecuentes se encuentran edades jóvenes, elevada demora para la obtención de la cita, bajo nivel socioeconómico o ausencia de recordatorio de la cita^12^^,^^13^. En Atención Primaria y en especialidades como Neurología o Urología, se han analizado los motivos de absentismo autorreferidos por los pacientes mediante entrevistas o encuestas telefónicas^6^^,^^14^^-^^16^. Sin embargo, en ORL existen pocos estudios que incorporen la perspectiva del paciente en el análisis de las causas de no asistencia^11^^,^^17^.

El presente estudio tiene como objetivo analizar los motivos de inasistencia a las consultas externas de ORL autorreferidos por los pacientes, clasificarlos en función de su potencial evitabilidad y cuantificar la proporción de consultas que son recitadas por el mismo motivo.

## MATERIAL Y MÉTODOS

Se realizó un estudio observacional retrospectivo en las consultas externas de ORL del Hospital Universitario de Torrevieja (España), hospital público de segundo nivel. El hospital pertenece a un departamento de salud que atiende a población costera y semiurbana, caracterizada por una proporción significativa de población extranjera y desplazada.

Se utilizó el sistema de información clínica y citaciones para identificar las consultas externas de ORL no atendidas de pacientes adultos y pediátricos durante el periodo comprendido entre el 1 de enero y el 31 de diciembre de 2024. Se definió la *consulta no atendida* como aquella a la que el paciente no acudió o canceló con menos de 24 horas respecto a la hora programada. Se consideró *recitación por el mismo motivo* cuando, tras el episodio de inasistencia, existía una consulta posterior registrada por causa similar.

Se incluyeron las consultas no atendidas correspondientes a pacientes con teléfono registrado en la historia clínica electrónica. El tamaño muestral se calculó sobre el total de 1.117 consultas no atendidas, asumiendo un nivel de confianza del 95%, un error máximo del 5% y una proporción esperada del 50% (criterio conservador ante la ausencia de datos previos), obteniéndose una n de 299 consultas.

La selección se realizó mediante muestreo aleatorio simple utilizando la función de selección aleatoria (IBM SPSS *Statistics* v.31.0). En caso de imposibilidad de contacto telefónico o negativa a participar, el caso no se sustituyó a fin de preservar la representatividad de la selección aleatoria inicial.

Los pacientes seleccionados (n=299) se contactaron telefónicamente entre febrero y junio de 2025. La recogida de datos se realizó mediante una entrevista telefónica estructurada de unos diez minutos de duración que incluía: presentación del investigador; información sobre el propósito del estudio y obtención del consentimiento verbal; identificación del motivo de no asistencia; registro de información complementaria abierta; y verificación, a través de la historia clínica, de la existencia de una reprogramación posterior por el mismo motivo de consulta.

Las entrevistas fueron realizadas por dos facultativos del servicio, previamente instruidos en el protocolo de recogida de datos. Se realizaron hasta dos intentos de contacto en días y en franjas horarias diferentes antes de clasificar el caso como *no contactado*.

Para cada paciente asociado a una consulta se recogieron de la historia clínica las siguientes variables demográficas y clínicas: edad (años), sexo (mujer, hombre), nacionalidad (española, extranjera), y nivel de cronicidad asignado según el modelo poblacional adoptado en la *Estrategia para la Atención a Pacientes Crónicos* en la Comunitat Valenciana^18^ (niveles 0 a 3).

De las consultas se recogieron el tipo de consulta (primera, sucesiva), prioridad (ordinaria, preferente), el mes, día y turno (mañana/tarde) de la citación. Los datos se obtuvieron de la herramienta corporativa *Cynara Intelligence* y el nivel de cronicidad del sistema PROSIGA de la *Consellería de Sanitat*.

Las variables resultado fueron:

- el motivo de inasistencia declarado por el paciente. Los motivos de inasistencia se clasificaron siguiendo una tipología previamente publicada y adaptada al presente estudio^6^ (Anexo I)**.** Se mantuvieron las diez categorías originales: error administrativo, fallo en la comunicación, pendiente de pruebas, demora excesiva, olvido, cambio de domicilio, mejoría clínica, insatisfacción con el servicio, otras prioridades e incapacidad física. Para este estudio, se añadió una adicional (*otro motivo*) para recoger razones de inasistencia no contempladas en la clasificación inicial, incluyendo aquellos pacientes que no recordaban el motivo de inasistencia. La asignación de los motivos se realizó según las definiciones preestablecidas de cada categoría.
- la asistencia posterior por el mismo motivo. Se consideró “sí” cuando se registró una nueva cita por la misma causa y “no” en ausencia de registro posterior.

Posteriormente, las categorías de inasistencia se agruparon en evitables o no evitables, según la capacidad potencial de intervención o de gestión por el sistema sanitario^6^^,^^19^, no desde la perspectiva del paciente. Las respuestas de la categoría *otro motivo* se revisaron individualmente y fueron reasignadas a uno de los grupos según su grado de evitabilidad.

Con el objetivo de identificar y agrupar patrones, las respuestas abiertas se sometieron a un análisis temático descriptivo, siguiendo un procedimiento de codificación inductiva realizada por un único investigador. Los códigos obtenidos se agruparon en categorías temáticas derivadas del contenido semántico y funcional de las respuestas. Posteriormente, las categorías resultantes se vincularon con las dimensiones del modelo conceptual de acceso a la atención sanitaria de Levesque y col^8^ (*approachability*, *acceptability*, *affordability*, *availability and accommodation* y *appropriateness*), con el propósito de identificar en qué fase del proceso operaba cada barrera. La asignación de cada categoría a su dimensión fue realizada de forma independiente por dos investigadores siguiendo los criterios operativos recogidos en el Anexo II.

### Análisis estadístico

Las variables cuantitativas se describieron mediante media y desviación estándar, mientras que las variables cualitativas se expresaron como frecuencias absolutas y porcentajes. Para la comparación entre grupos se emplearon pruebas no paramétricas para la variable continua edad, y la prueba Chi-cuadrado (*χ^2^*) para las categóricas, utilizando la prueba exacta de Fisher cuando alguna frecuencia esperada fue inferior a 5. Se exploró la asociación entre la clasificación de los motivos de inasistencia (evitables/no evitables) y las variables sexo y nacionalidad mediante Chi-cuadrado; la comparación de edad según evitabilidad se realizó mediante U de Mann-Whitney. Todos los cálculos se realizaron con SPSS v.31.0 (IBM Corp, Armonk, NY, EEUU). Se consideró un nivel de significación de *p* < 0,05 en contraste bilateral.

## RESULTADOS

La muestra seleccionada fueron 299 consultas correspondientes a 292 pacientes. Siete pacientes registraron dos episodios de consultas no atendidas; no obstante, cada consulta se consideró de forma independiente conforme a la unidad de análisis predefinida.

**Figura 1:**
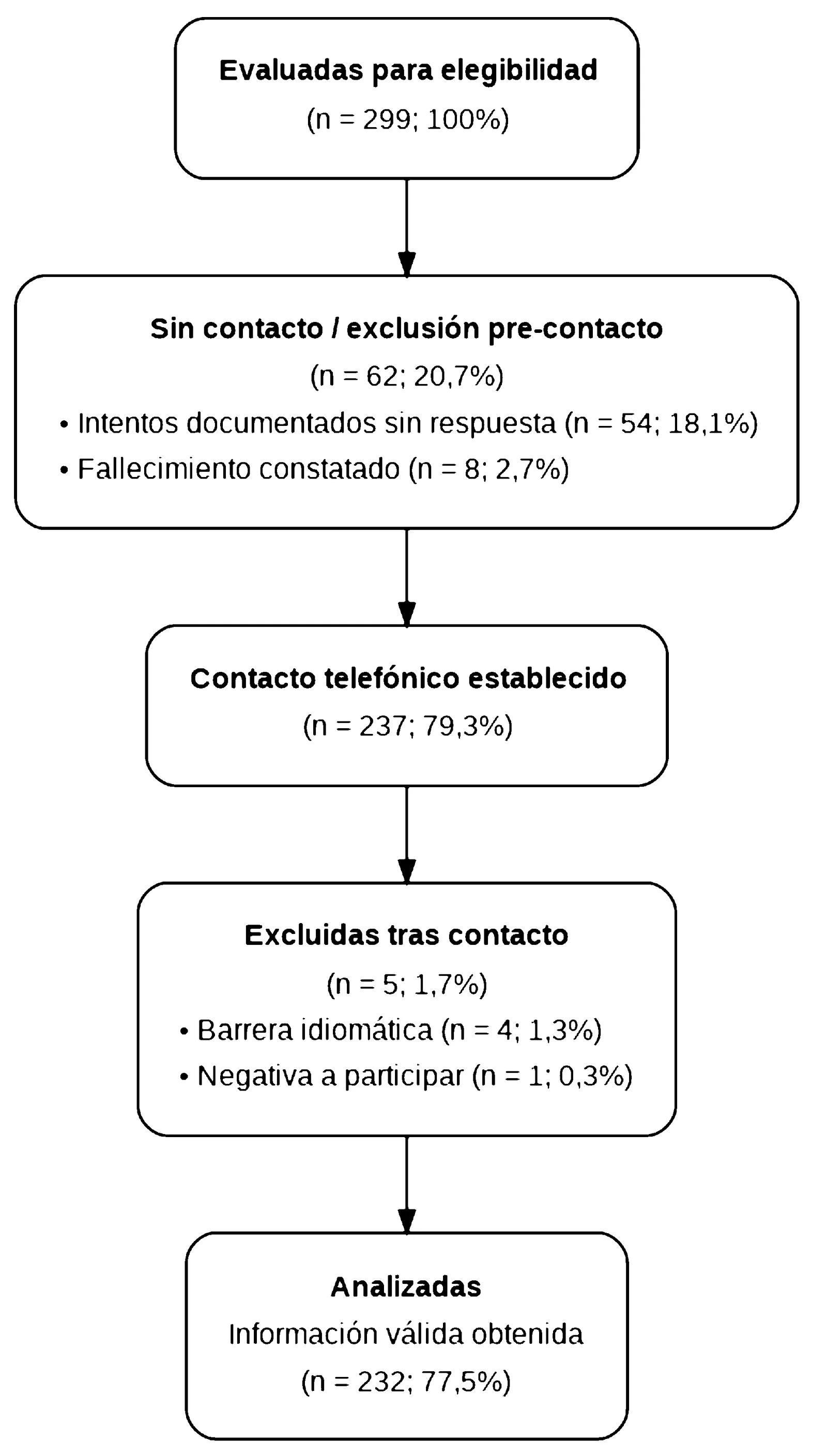
Características del proceso de selección de la muestra analizada.

Ocho pacientes se excluyeron por fallecimiento. Se estableció contacto telefónico en relación a 237 consultas (79%). Cuatro pacientes se excluyeron por barrera idiomática y uno por negativa a participar, por lo que la muestra final quedó constituida por 232 consultas, que representa el 77,5% de la muestra inicial (Fig. 1).

La muestra analizada mostró un ligero predominio de varones (52,2%) y de personas con nacionalidad española (53,0%); la mediana de edad fue de 45,5 años (RIC=29-62) (Tabla 1). Más de la mitad de los pacientes se encontraban en el nivel 1 de nivel de cronicidad (51,3%) y el 27,2% en el nivel 0 (Tabla 1).

**Tabla 1:** Características sociodemográficas y clínico-administrativas de la muestra entrevistada (n=232)

Variable	n (%)
*Persona*	
*Edad (años)*	
Media (DE)	45,4 (21,1)
Mediana (RIC)	45,5 (29-62)
Sexo (masculino)	121 (52,2)
Nacionalidad (española)	123 (53,0)
*Cronicidad*	
Nivel 0	63 (27,2)
Nivel 1	119 (51,3)
Nivel 2	36 (15,5)
Nivel 3	14 (6,0)
*Consulta no asistida*	
Tipo (sucesiva)	223 (96,1)
Prioridad (ordinaria)	215 (92,7)
*Mes*	
Enero	25 (10,8)
Febrero	14 (6,0)
Marzo	26 (11,2)
Abril	22 (9,5)
Mayo	20 (8,6)
Junio	14 (6,0)
Julio	14 (6,0)
Agosto	14 (6,0)
Septiembre	17 (7,3)
Octubre	20 (8,6)
Noviembre	25 (10,8)
Diciembre	21 (9,1)
*Día de la semana*	
Lunes	37 (15,9)
Martes	60 (25,9)
Miércoles	68 (29,3)
Jueves	48 (20,7)
Viernes	19 (8,2)
*Turno*	
Mañana	206 (88,8)
Tarde	26 (11,2)

DE: desviación estándar; RIC: rango intercuartílico.

Solo el 3,9% de las citas correspondían a primeras consultas y el 7,3% tuvieron carácter preferente. La distribución mensual de citas no atendidas fue relativamente homogénea a lo largo del año, con enero, marzo y noviembre por encima del 10% y febrero, junio, julio y agosto con un 6%. Por día de la semana, el miércoles concentró el mayor número de ausencias (29,3%) y el viernes el menor (8,2%) (Tabla 1).

El fallo en la comunicación fue el motivo de absentismo más frecuente reportado por los pacientes (32,3%), seguido por otras prioridades personales o laborales (19,4%) y *otros motivos* (16,8%). Las causas menos prevalentes (<3%) fueron el cambio de domicilio, la insatisfacción con el servicio y el error administrativo (Tabla 2). Los motivos de inasistencia no se relacionaron con el sexo (χ^2^_9_=7,685; p=0,566) ni la nacionalidad (χ^2^_9_=9,148; p=0,424). En ambos casos el 40% de las casillas presentaron frecuencias esperadas pequeñas, lo que aconseja interpretar los resultados con cautela.

**Tabla 2:** Motivos declarados de absentismo en las consultas externas de Otorrinolaringología (n = 232)

Motivo de absentismo	n (%)	Clasificación de evitabilidad
Fallo en la comunicación	75 (32,3)	Evitable
Otras prioridades	45 (19,4)	No evitable
Otros motivos	39 (16,8)	No evitable
Incapacidad física	23 (9,9)	No evitable
Olvido	18 (7,8)	Evitable
Mejoría clínica	17 (7,3)	No evitable
Cambio de domicilio	6 (2,6)	No evitable
Insatisfacción con el servicio	4 (1,7)	No evitable
Error administrativo	3 (1,3)	Evitable
Demora excesiva	2 (0,9)	Evitable
Total	232 (100)	

Dentro de *otros motivos*, el 66,7% de pacientes no recordaban el motivo; otras causas fueron rechazo al tratamiento, dificultades de transporte, incidencias administrativas externas y causas misceláneas. Según los criterios preestablecidos, todas las causas agrupadas en esta subcategoría se clasificaron como no evitables (Tabla 3).

**Tabla 3:** Motivos declarados de absentismo en las consultas externas de Otorrinolaringología clasificados como *otros motivos* (n = 39)

Subcategoría	Definición operativa	n	% (n=39)	% (n=232)
No recuerda	Ausencia de recuerdo del motivo	26	66,7	11,2
Rechazo terapéutico	Rechazo de tratamiento o pruebas	5	12,8	2,2
Falta de transporte/apoyo	Dificultad de desplazamiento	3	7,7	1,3
Problemas administrativos externos	Incidencias no hospitalarias	2	5,1	0,9
Otros (miscelánea)	Situaciones no clasificables	3	7,7	1,3
Total		39	100	16,8

Globalmente, y atendiendo a su potencial evitabilidad, 98 consultas (42,2%) asociaron su inasistencia a causas evitables; el fallo en la comunicación y el olvido de la cita fueron las más frecuentes (Tabla 4). No se identificaron asociaciones estadísticamente significativas entre la evitabilidad y el sexo (41,3% en hombres frente a 43,2% en mujeres; p=0,767), la nacionalidad (40,3% española frente a 44,4% extranjera; p=0,526) ni la mediana de edad (47; RIC: 30-64 frente a 45; RIC: 29-62; p=0,336).

Tras la inasistencia, se reprogramó una consulta posterior en el 25,0% (n=58) de los casos. No se identificaron asociaciones estadísticamente significativas con el sexo (25,6% en hombres frente a 24,3% en mujeres; p=0,820), la nacionalidad (26,6% española frente a 23,1% extranjera; p=0,543) o la mediana de edad (47; RIC: 30-64 frente a 45; RIC: 28-62; p=0,949).

El análisis descriptivo de las respuestas abiertas incluyó una codificación temática cuya correspondencia con el modelo Levesque se presenta en la tabla 4. Dado que una misma respuesta podía recibir múltiples códigos, se identificaron 277 códigos en las 232 respuestas. Tras el proceso de agrupación emergieron 20 categorías temáticas. La categoría más frecuente fue *problemas de comunicación con la cita* (22,0%), seguida de lejos por *desconocimiento del motivo de la inasistencia*, *barreras laborales* y *mejoría clínica* (<10%).

El análisis de los motivos de inasistencia según el modelo de acceso de Levesque y col^8^ se realizó sobre las 19 categorías temáticas clasificables, excluyendo la categoría “El paciente no se acuerda” (n=26) por constituir un artefacto metodológico atribuible al sesgo de recuerdo. El total de menciones clasificables fue de 251. La dimensión *approachability* fue la más frecuente (44,6%), seguida de *appropriateness* (29,5%) y *availability and accommodation* (20,3%). Las dimensiones *acceptability* (4,8%) y *affordability* (0,8%) presentaron las frecuencias más bajas.

**Tabla 4:** Categorías temáticas de los motivos de inasistencia a consultas externas de Otorrinolaringología derivadas de las respuestas abiertas y su correspondencia con el modelo de acceso a la atención sanitaria de Levesque

Categoría temática	Descripción	n (%)	Ejemplo textual	Dimensión (Levesque)
Problemas de comunicación en la cita	El paciente no recibió la notificación de la cita o la desconocía	61 (22,0)	“No sabía que tenía cita”	*Approachability*
Mensaje	Fallo atribuible específicamente al recordatorio por SMS	28 (10,1)	“No recibí el mensaje”	*Approachability*
El paciente no se acuerda	Incapacidad para recordar el motivo de la inasistencia debido al tiempo transcurrido entre la cita y la entrevista	26 (9,4)	“No recuerdo”	No clasificable
				(sesgo de recuerdo)
Barreras laborales o de horario	Incompatibilidad entre la jornada laboral y la cita	25 (9,0)	“Soy camionero”	*Availability and Accommodation*
			“Iba liado con el trabajo”	
Mejoría clínica percibida	Resolución o mejoría sustancial de los síntomas	24 (8,7)	“Ya estaba bien”	*Appropriateness*
Enfermedad o incapacidad física para acudir	Enfermedad intercurrente o limitación física que imposibilitó la asistencia.	23 (8,3)	“Tenía gripe”	*Appropriateness*
			“Estaba recién operada”	
Olvido explícito	Olvido de la cita pese a conocerla.	16 (5,8)	“Se me pasó”	*Approachability*
Extranjero	Traslado temporal o definitivo al extranjero	10 (3,6)	“Estaba en Bélgica”	*Availability and Accommodation*
Necesidad de cita o seguimiento	Persistencia o reaparición de síntomas en el momento de la entrevista, con el paciente expresando necesidad de valoración clínica	11 (4,0)	“Me gustaría una nueva cita	*Appropriateness*
Baja priorización	Inasistencia basada en una valoración subjetiva de baja prioridad de la cita, sin restricción externa.	11 (4,0)	“Tenía un viaje”	*Acceptability*
			“Iba liado con el trabajo”	
Disconformidad o rechazo terapéutico	Rechazo del tratamiento o procedimiento diagnóstico propuesto, o insatisfacción con la atención previa recibida.	9 (3,2)	“No quería hacer ningún tratamiento”	*Appropriateness*
Cambio de cita no confirmado	Intento de cancelación o cambio de cita sin registro en el sistema	7 (2,5)	“Llamé para anular”	*Approachability*
Centro privado	Uso electivo de la atención sanitaria privada	6 (2,2)	“Me fui por privado”	*Appropriateness*
Factores sociales o familiares	Obligaciones de cuidado o acontecimiento familiar urgente que impidieron la asistencia.	6 (2,2)	“Falleció un familiar”	*Availability and Accommodation*
Barreras de transporte	Imposibilidad de desplazarse al centro por ausencia de vehículo propio o acompañante.	5 (1,8)	“No tenía coche”	*Availability and Accommodation*
Otra ciudad	Estancia temporal en otra ciudad	4 (1,4)	“Vivo en Madrid”	*Availability and Accommodation*
Motivos económicos	Dificultades económicas o de cobertura	2 (0,7)	“Problemas con el seguro”	*Affordability*
Barreras culturales o idiomáticas	Dificultades culturales o lingüísticas	1 (0,4)	“Quería un médico hombre”	*Acceptability*
Demora	Búsqueda de atención alternativa por demora.	1 (0,4)	“Me fui por privado”	*Appropriateness*
Retraso o incidencia en el tráfico	El paciente se desplazó, existiendo una incidencia imprevista	1 (0,4)	“Llegué tarde”	*Availability and Accommodation*

## DISCUSIÓN

El hallazgo principal de este estudio fue que el fallo en la comunicación constituyó el motivo de inasistencia más frecuente, representando casi un tercio de todos los episodios analizados, lo que posiciona los factores organizativos del sistema como impulsores primarios del absentismo en este contexto. Su frecuencia puede variar según el contexto organizativo y el sistema de citación, por lo que algunos estudios lo identifican como la causa más frecuente^14^^,^^20^, mientras que otros lo sitúan en segundo lugar, después del olvido de la cita^3^^,^^21^.

No obstante, la distribución del conjunto de motivos declarados (con hasta diez categorías identificadas) confirma que el absentismo en consultas externas es un fenómeno multifactorial. Comprender este fenómeno en toda su complejidad requiere marcos conceptuales sólidos que vayan más allá de describir el hecho de asistir o no, a fin de identificar los mecanismos responsables subyacentes. Ejemplos de estos marcos teóricos son el propuesto por Penchansky y Thomas^22^, o el de Levesque y col^8^ empleado en el presente estudio.

Según una revisión de la literatura, el olvido de la cita emerge como uno de los motivos más frecuentes de absentismo^23^. Sin embargo, en el presente estudio ocupa el cuarto lugar. Esta discrepancia podría explicarse por varios factores: las diferencias en el contexto organizativo y en la modalidad del sistema de citación empleado, así como por la disponibilidad de sistemas de recordatorio activo^24^. Sin embargo, el diseño observacional del estudio no permite establecer inferencias causales.

La relegación de la cita por compromisos laborales o personales fue el segundo motivo más frecuente de absentismo, en concordancia con otros estudios, que identifican la incompatibilidad entre los horarios laborales y el horario de la cita como uno de los factores más relevantes asociados a la inasistencia^21^^,^^25^^,^^26^. Esta barrera de naturaleza estructural más que individual, apunta a la necesidad de implementar modelos asistenciales más flexibles, con ampliación de horarios o incluso modalidad de asistencia no presencial/telemedicina. No obstante, su puesta en marcha puede incluso aumentar el absentismo durante las fases iniciales de adaptación, lo que subraya la importancia de acompañar cualquier cambio organizativo de estrategias específicas de comunicación y acompañamiento al paciente^27^.

La clasificación de los motivos de absentismo en evitables y no evitables mostró que aproximadamente el 60% correspondieron a motivos no evitables, resultado coherente con estudios previos^3^^,^^6^. No obstante, esta categoría no debería entenderse como sinónimo de inevitabilidad absoluta: numerosas causas no evitables podrían estar condicionadas por una rigidez organizativa del sistema sanitario (horarios, canales de comunicación), más que por circunstancias exclusivamente personales del paciente^25^^,^^28^.

Solo una cuarta parte de las consultas derivaron en una nueva cita por el mismo motivo, cifra que contrasta con el 50% descrito en estudios previos^3^. Esta diferencia podría reflejar, en parte, la ausencia de un sistema de reprogramación automática en el servicio. Esto traslada al paciente una mayor responsabilidad sobre la gestión de su atención y se aparta de modelos asistenciales de corte paternalista^29^. No obstante, la ausencia de reprogramación sistemática exige la identificación proactiva de los pacientes más vulnerables, dado que el absentismo en personas con patología crónica o condicionantes sociales se ha asociado con un mayor riesgo de morbimortalidad^30^.

La tasa de contacto efectivo fue alta (en torno al 80%) y comparable a la descrita por otros autores^26^, pese a que el paciente pueda percibir la investigación sobre el absentismo como una intromisión incómoda. Asimismo, la tasa de rechazo a participar (un único paciente: 0,3%) fue notablemente inferior al 7% registrado en un estudio sobre absentismo con 316 participantes^31^.

La interpretación de los resultados a través del modelo de acceso de Levesque y col^8^ permite identificar que una parte sustancial del absentismo se origina en fases tempranas del proceso de citación, lo que sitúa la inasistencia como expresión de un desajuste entre la oferta del sistema y las necesidades del paciente en etapas iniciales. El predominio de la dimensión *approachability* es coherente con los hallazgos de Aldadi y col, quienes identifican fallos en la comunicación (recordatorios no leídos o extraviados, información inadecuada, problemas de programación) como determinantes en el absentismo que actúan principalmente desde el lado de la oferta del sistema^7^. La segunda dimensión en importancia fue *appropriateness*, que agrupa situaciones relacionadas con la mejoría clínica previa a la consulta, la incapacidad física o la búsqueda de atención alternativa, factores que expresan las necesidades del paciente^7^.

Estudios cualitativos sugieren que un tercio de las no asistencias son atribuibles a fallos de comunicación institucional, y no a decisiones deliberadas del propio paciente^21^, lo que cuestiona el enfoque tradicional que atribuye la responsabilidad del absentismo exclusivamente al paciente. Por lo tanto, el absentismo no debería interpretarse como una conducta individual, sino como un indicador de la capacidad del sistema para adaptarse a las circunstancias del paciente^8^. En consecuencia, las intervenciones orientadas a reducir las tasas de absentismo deberían priorizar la flexibilidad organizativa y los canales de comunicación con el servicio, en lugar de orientarse exclusivamente a modificar la conducta del paciente^19^^,^^25^.

Los sistemas sanitarios podrían beneficiarse de estrategias de recordatorio de citaciones multicanal y estratificadas por perfil de paciente^32^^,^^33^. Aunque los mensajes automatizados han demostrado ser una herramienta eficaz, sobre todo en gente joven y con adecuadas competencias digitales^33^, no deberían sustituir a la llamada telefónica personalizada, que puede ser más efectiva en personas mayores dado que la recepción de un mensaje no garantiza su lectura^32^. Facilitar canales de confirmación, cancelación o reprogramación de la cita podría constituir una estrategia prioritaria.

Entre las limitaciones de este estudio destaca el sesgo de recuerdo (el 11,2% de los entrevistados no recordaban el motivo por el que habían faltado a la consulta), derivado del intervalo de tiempo transcurrido entre el episodio de inasistencia y la entrevista. Asimismo, las entrevistas realizadas por personal facultativo del mismo servicio podrían haber inducido un sesgo de respuesta, inclinando al paciente a formular respuestas más aceptables socialmente^34^. La codificación temática fue realizada por un único investigador, lo que constituye una limitación en términos de fiabilidad interevaluador; los hallazgos deben interpretarse, por tanto, con cautela y no como resultado de un análisis sistemático de contenido. Respecto al marco teórico de Levesque y col^8^, si bien ha demostrado su utilidad en estudios de accesibilidad y uso de servicios sanitarios^9^^,^^35^, su aplicación específica al análisis del absentismo es limitado^7^, lo que dificulta la comparación de los resultados. La ausencia de comparación de las características clínicas y sociodemográficas de los pacientes que acuden a sus citas frente a los que no acuden no permite identificar factores predictores del absentismo, que complementarían los hallazgos descriptivos del presente estudio.

Entre las fortalezas del estudio destaca, en primer lugar, la incorporación de la perspectiva autorreferida por el paciente en las consultas externas de ORL, donde la literatura se ha apoyado tradicionalmente en datos administrativos. A ello se suma el muestreo aleatorio, la alta tasa de respuesta a la entrevista y el uso de la consulta como unidad de análisis, pertinente desde el punto de vista organizativo. Finalmente, la integración de un análisis descriptivo estructurado con el marco conceptual de Levesque es una aproximación novedosa y permite reinterpretar la inasistencia como un proceso asistencial con un itinerario propio, en el que es posible identificar momentos críticos susceptibles de intervención. Además, la utilización de dos enfoques analíticos en un mismo estudio refuerza la coherencia interna de los hallazgos.

En conclusión, el absentismo en las consultas externas de ORL se vinculó principalmente a aspectos organizativos y comunicativos durante las etapas tempranas del proceso de citación, que no aseguran que el paciente reciba, comprenda adecuadamente y retenga en su memoria la información de la cita. El 42,2% de las inasistencias se identificaron como potencialmente evitables mediante intervenciones organizativas específicas. No obstante, el absentismo no generó una demanda de recitación equivalente, dado que solo el 25% de los pacientes solicitó una nueva cita.

Los hallazgos de este estudio pueden constituir un punto de partida para desarrollar estrategias efectivas por parte de las organizaciones, orientadas a flexibilizar al sistema, mejorar los canales de comunicación y reducir las barreras tempranas de acceso.

## Data Availability

Se encuentran disponibles bajo petición al autor de correspondencia.

## ANEXO I

### Categorías de motivos de no asistencia, definiciones operativas y capacidad de intervención institucional

**Table:**

Categoría	Definición operativa	Capacidad de intervención institucional
Error administrativo	Incidencia en la gestión de la cita atribuible al sistema.	Evitable
Fallo en la comunicación	La información de la cita no fue recibida o comprendida correctamente por el paciente.	Evitable
Pendiente de pruebas	La consulta no se realizó por ausencia de resultados diagnósticos necesarios en el momento de la cita.	Evitable
Demora excesiva	Intervalo de espera percibido como excesivo entre la citación y la fecha de consulta.	Evitable
Olvido	No asistencia atribuida al olvido de la cita por parte del paciente pese a conocer su existencia.	Evitable
Cambio de domicilio	Traslado de residencia que impidió la asistencia a la consulta.	No evitable
Mejoría clínica	No asistencia por resolución o mejoría de los síntomas.	No evitable
Insatisfacción con el servicio	No asistencia relacionada con descontento previo con la atención o con el servicio sanitario.	No evitable
Otras prioridades	Relegación de la consulta por conflictos laborales o personales.	No evitable
Incapacidad física	Limitación física o enfermedad intercurrente que impidió el desplazamiento.	No evitable
Otro motivo	Motivo no clasificable en las categorías anteriores según la respuesta del paciente.	Variable

Clasificación basada en la propuesta por Morera-Guitart y col^6^, con la incorporación de la categoría “Otro motivo”, adaptada al presente estudio. La clasificación de evitabilidad se realizó desde la perspectiva de capacidad de intervención institucional.

### ANEXO II

Definición operativa de las cinco dimensiones del modelo de acceso de Levesque et al. (2013)^8^ para la codificación de categorías temáticas

**Table:**

Dimensión	Definición operativa	Criterio de asignación
*Approachability*	Categorías cuyo núcleo es un fallo en la recepción, comprensión o retención de la información de la cita. La barrera actúa antes de que el paciente pueda tomar ninguna decisión: no sabe que tiene cita, la olvida o cree haberla gestionado sin que quedara registro.	La categoría describe:
Capacidad de percibir		(a) no recepción del aviso, (b) incomprensión del mensaje,
		(c) olvido de la cita una vez conocida,
		(d) pérdida del recuerdo por el paso del tiempo, o
		(e) gestión de cancelación sin registro en el sistema.
*Acceptability*	Categorías en las que el paciente conocía la cita y podía acudir, pero tomó la decisión voluntaria de no ir, bien por baja valoración subjetiva de su necesidad de atención, bien por una barrera cultural o idiomática que condicionó su disposición a buscar ayuda.	La categoría describe una decisión discrecional no condicionada por ningún impedimento externo concreto, o una preferencia cultural/idiomática que actuó como filtro en la búsqueda de atención.
Capacidad de buscar		
*Availability and Accommodation*	Categorías en las que el paciente quería acudir, pero no pudo estar físicamente presente. La barrera es externa: incompatibilidad horaria laboral, falta de transporte o acompañante, ausencia geográfica, obligaciones familiares o una incidencia durante el trayecto.	La categoría describe un impedimento físico o circunstancial externo que impidió que el paciente llegara al punto de atención, independientemente de su disposición a acudir.
Capacidad de alcanzar		
*Affordability*	Categorías en las que el paciente menciona explícitamente una dificultad económica como causa de la inasistencia: coste del transporte, pérdida de ingresos por ausentarse del trabajo o problemas con la cobertura del seguro sanitario.	La categoría describe una barrera de tipo económico, ya sea por coste directo (transporte, parking) o indirecto (pérdida de jornal, seguro). La mención explícita del factor económico es el criterio diferenciador respecto a L3.
Capacidad de pagar		
*Appropriateness*	Agrupa las categorías en las que el paciente evalúa si la cita seguía siendo pertinente para su estado de salud: mejoría de síntomas, enfermedad intercurrente, rechazo del tratamiento, búsqueda de alternativas o preferencia por atención privada. Incluye también la expresión de necesidad persistente de atención.	La categoría describe una valoración del paciente sobre la adecuación clínica de la consulta, la pertinencia del tratamiento o la búsqueda de un recurso alternativo considerado más apropiado.
Capacidad de comprometerse		
